# Zinc Overload Enhances APP Cleavage and Aβ Deposition in the Alzheimer Mouse Brain

**DOI:** 10.1371/journal.pone.0015349

**Published:** 2010-12-17

**Authors:** Chun-Yan Wang, Tao Wang, Wei Zheng, Bao-Lu Zhao, Gorm Danscher, Yu-Hua Chen, Zhan-You Wang

**Affiliations:** 1 Key Laboratory of Cell Biology of Ministry of Public Health, and Key Laboratory of Medical Cell Biology of Ministry of Education, China Medical University, Shenyang, China; 2 State Key Laboratory of Brain and Cognitive Sciences, Institute of Biophysics, Academia Sinica, Beijing, China; 3 Department of Anatomy and Neurobiology, University of Aarhus, Aarhus, Denmark; Mental Health Research Institute of Victoria, Australia

## Abstract

**Background:**

Abnormal zinc homeostasis is involved in β-amyloid (Aβ) plaque formation and, therefore, the zinc load is a contributing factor in Alzheimer's disease (AD). However, the involvement of zinc in amyloid precursor protein (APP) processing and Aβ deposition has not been well established in AD animal models *in vivo*.

**Methodology/Principal Findings:**

In the present study, APP and presenilin 1 (PS1) double transgenic mice were treated with a high dose of zinc (20 mg/ml ZnSO4 in drinking water). This zinc treatment increased APP expression, enhanced amyloidogenic APP cleavage and Aβ deposition, and impaired spatial learning and memory in the transgenic mice. We further examined the effects of zinc overload on APP processing in SHSY-5Y cells overexpressing human APPsw. The zinc enhancement of APP expression and cleavage was further confirmed *in vitro*.

**Conclusions/Significance:**

The present data indicate that excess zinc exposure could be a risk factor for AD pathological processes, and alteration of zinc homeostasis is a potential strategy for the prevention and treatment of AD.

## Introduction

The presence of extracellular β-amyloid (Aβ) plaques in the brain is one of the pathological hallmarks of Alzheimer's disease (AD). Mounting evidence has demonstrated that aberrant zinc homeostasis is involved in the pathogenesis of AD [1], [2], [3], [4]. In the post-mortem AD brain, a marked accumulation of zinc is found in the Aβ plaques [5], [6], [7], [8], [9], [10]. Since Aβ peptide has zinc-binding sites, and zinc is the only physiologically available metal able to precipitate Aβ, the abnormal enrichment of zinc in the AD brain indicates that zinc binding to Aβ plays a role in the formation of amyloid plaques [11]. Furthermore, zinc chelating agents, such as clioquinol (CQ) and DP-109, that modulate brain zinc levels can inhibit the formation of amyloid plaques [12], [13], [14]. In preliminary studies, CQ has shown some effects on cognition in AD patients [15], [16], [17]. Thus, abnormal zinc homeostasis is believed to be a contributing factor leading to Aβ aggregation, and alteration of zinc homeostasis is a potential therapeutic strategy for AD.

The disruption of zinc homeostasis in the AD brain is associated with the aberrant distribution and altered expression of zinc-regulating metalloproteins, such as metallothionein, zinc transporters (ZnT) and divalent metal transporter 1 (DMT1). We have reported that high levels of ZnT1, 3-7 and DMT1 proteins are located in the degenerating neurites in or around the Aβ-positive plaques associated with human AD and the APP/presenilin 1 (PS1) transgenic mouse brain [18], [19], [20], [21]. Significant alterations in the expression levels of ZnT1, 4, and 6 have been detected in AD postmortem brain specimens [22], [23]. Genetic abolition of ZnT3 results in disappearance of zinc ions in the synaptic vesicles [24], and leads to an age-dependent deficit in learning and memory in ZnT3 knockout mice [25]. Most interestingly, a markedly reduced plaque load and less insoluble Aβ have been observed in ZnT3 knockout plus APP overexpressed mouse brain [26], suggesting a role of synaptic zinc in Aβ generation and aggregation. Furthermore, *in vitro* studies have shown that both APP and its proteolytic product Aβ contain zinc binding domains. However, the involvement of zinc in APP processing and Aβ deposition has not been well established in AD transgenic models *in vivo*.

In the present study, we extended our experiments to examine whether chronic intake of water containing a high level of zinc accelerates Aβ deposition and APP cleavage in APP/PS1 mouse brain. We found that a high level of dietary zinc could cause cognition dysfunction and enhance the aggregation of Aβ. Furthermore, we found that a high level of zinc also enhanced Aβ generation through altering the expression levels of APP and APP cleavage enzymes *in vivo* and *in vitro*. Our data support the possibility that dietary zinc overload has the potential to be a contributing factor to the pathophysiology of AD.

## Results

### Chronic high intake of dietary zinc induces spatial learning-memory deficits in APP/PS1 mice

**Figure 1:**
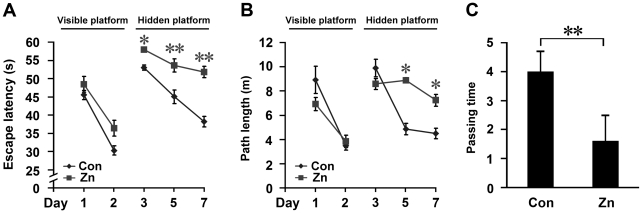
Morris water maze assessment of APP/PS1 transgenic mice. APP/PS1 mice at the age of 3 months were given either a standard diet and deionized water (Con), or a standard diet and deionized water containing 20 mg/ml ZnSO4 (Zn). Morris water maze tests were performed to evaluate whether high dietary zinc treatment affects learning and memory in APP/PS1 mice at the age of 9 months. (**A**, **B**) In the visible platform training from day 1 to 2, mice in different groups exhibited a similar escape latency and path length to find the visible platform. At day 3, 5, and 7 of the hidden platform tests, Zn-treated mice showed the longest latency and escape length. (**C**) In the probe trial on the last day, the Zn-treated mice exhibited the lowest passing times into the northwest quadrant, where the hidden platform was previously located. * *p*<0.05, ** *p*<0.01 versus control group (repeated measures ANOVA).

APP/PS1 transgenic mice at the age of 3 months were given a standard diet and deionized water containing ZnSO_4_ (20 mg/ml). Morris water maze tests were performed to evaluate whether high dietary zinc treatment affects learning and memory in APP/PS1 mice at the age of 9 months. These included 2 days of visible platform training, 5 days of hidden platform tests, and a probe trial 1 day after the last hidden platform test (Figure 1). The visible platform tests showed that the zinc group and control mice had a similar escape latency and path length (*p*>0.05; Figure 1A, B), suggesting that zinc treatment did not significantly affect motility or vision in the transgenic mice. In the place navigation (hidden platform) tests, the zinc group mice showed a longer escape latency and a longer path length before swimming onto the hidden platform compared with the control mice fed a normal diet (*p*<0.01; Figure 1A, B). Furthermore, the probe trial showed that the number of times the mice traveled into the center of the northwest quadrant, where the hidden platform was previously placed, was significantly less for zinc group mice compared with controls (*p*<0.01; Figure 1C). Taken together, these data suggest that high-dose oral zinc leads to spatial learning-memory impairments in APP/PS1 mice.

### Enhancement of zinc content in serum and brain of APP/PS1 mice fed a zinc diet

**Figure 2:**
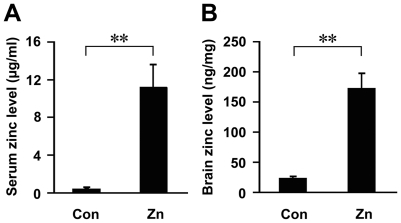
Changes in zinc level in APP/PS1 mice fed a zinc diet. (**A**) At the age of 9 months, the Zn group mice showed an increased level of serum zinc. (**B**) Brain zinc levels were also significantly increased in the Zn group mice. ** *p*<0.01 versus control group (Student's *t* test).

The serum zinc levels were measured in the transgenic mice at the age of 9 months. There was a significant increase in zinc level in the zinc group (11.21±2.42 µg/ml), compared with the control (0.43±0.12 µg/ml) (*p*<0.01; Figure 2A). We also measured the zinc level in the brain of the transgenic mice to determine the effects of a high zinc diet. The brain zinc level was 173.17±24.72 ng/mg in the zinc group, and 23.59±2.31 ng/mg in the control group. Statistical analysis showed that treatment with a high dose of zinc significantly increased the level of zinc in the brain (*p*<0.01; Figure 2B).

### Increase in the number of zinc-containing plaques in the brain of APP/PS1 mice fed a high zinc diet

**Figure 3:**
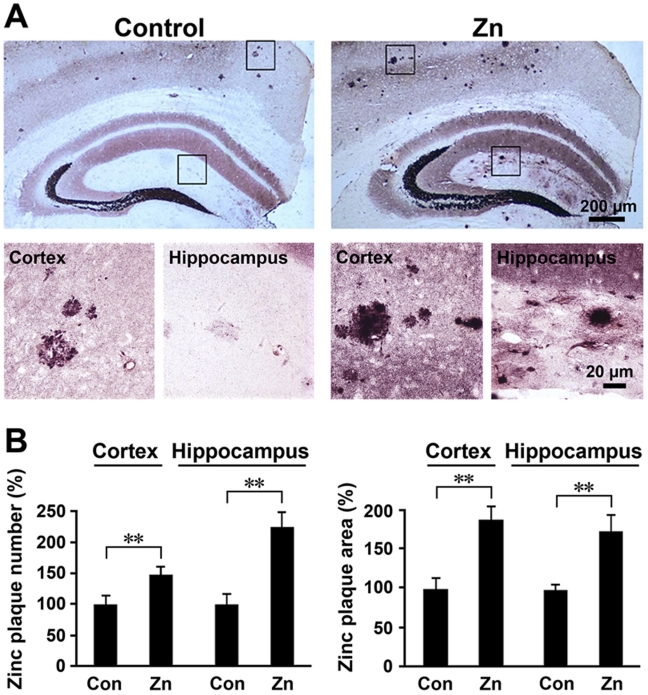
Zinc accumulation in neuritic plaques in APP/PS1 mice. (**A**) Zinc ions stained by AMG showing the distribution patterns of zinc ions in the cortex and hippocampus, especially mossy fibers. Importantly, the number and size of the zinc-containing plaques were increased in Zn-treated mice. (**B**) Statistical analysis showing that the number and size of zinc-containing neuritic plaques were significantly increased in Zn-treated mice compared with controls. ** *p*<0.01 versus control group (Student's *t* test).

The autometallography (AMG) procedure allows demonstration of a striking condensation of ionic zinc within plaques in the human postmortem AD brain and the APP/PS1 transgenic mouse brain [10], [19]. Brain sections of APP/PS1 mice given a high dose of zinc in their drinking water and normal diet were subjected to AMG analysis. In general, zinc-positive plaques were distributed throughout the cortex and hippocampus in all examined animals (Figure 3A), as previously described [27]. Both the number and size of the zinc-positive plaques in the zinc-treated group were markedly increased in the cortex and hippocampus (Figure 3A). Statistical analyses showed that high zinc treatment significantly increased the number of zinc-positive plaques by 146.24±12.30% in the cortex and 225.00±22.97% in the hippocampus respectively, compared with the control group (*p*<0.01; Figure 3B). The size of zinc-containing plaques in the brain of APP/PS1 mice fed a high zinc diet was increased by 186.83±15.74% in the cortex and 179.60±21.74% in the hippocampus, compared with the control group (*p*<0.01; Figure 3B).

### High dietary zinc increases Aβ burden in the APP/PS1 mouse brain

**Figure 4:**
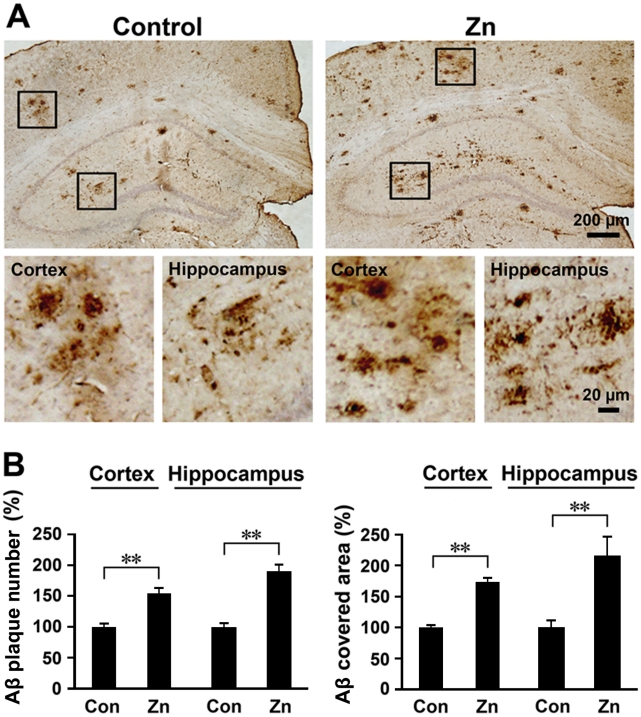
Zinc treatment enhances neuritic plaque formation in APP/PS1 mice. (**A**) Aβ immunohistochemical images showing the Aβ-positive plaques in the transgenic mouse brain. There were more neuritic plaques in Zn-treated mice compared with controls. (**B**) Quantification of neuritic plaques showed that both the number and size of neuritic plaques were increased in Zn-treated mice compared with controls. ** *p*<0.01 versus control group (Student's *t* test).

To determine whether chronic high intake of dietary zinc potentiates Aβ deposition, brain sections of APP/PS1 mice were subjected to Aβ immunohistochemical analysis. As shown in Figure 4, both the number and size of the Aβ-immonoreactive senile plaques were markedly increased in the cortex and hippocampus in the brain of zinc-treated mice (Figure 4A). Statistical analyses showed that a high intake of dietary zinc significantly increased the number of Aβ plaques by 154.55±8.25% in the cortex and 188.31±11.90% in the hippocampus, compared with the control group fed a normal diet (*p*<0.01; Figure 4B). We also evaluated the changes in Aβ burden by measuring the areas of Aβ-positive neuritic plaques in the mouse brain. In the zinc group, the area of Aβ plaques was significantly increased by 173.36±11.44% in the cortex and 213.15±34.29% in the hippocampus, compared with the control group (*p*<0.01; Figure 4B).

### Increase in expression level of APP protein and Aβ generation in the APP/PS1 mouse brain after a high zinc diet

**Figure 5:**
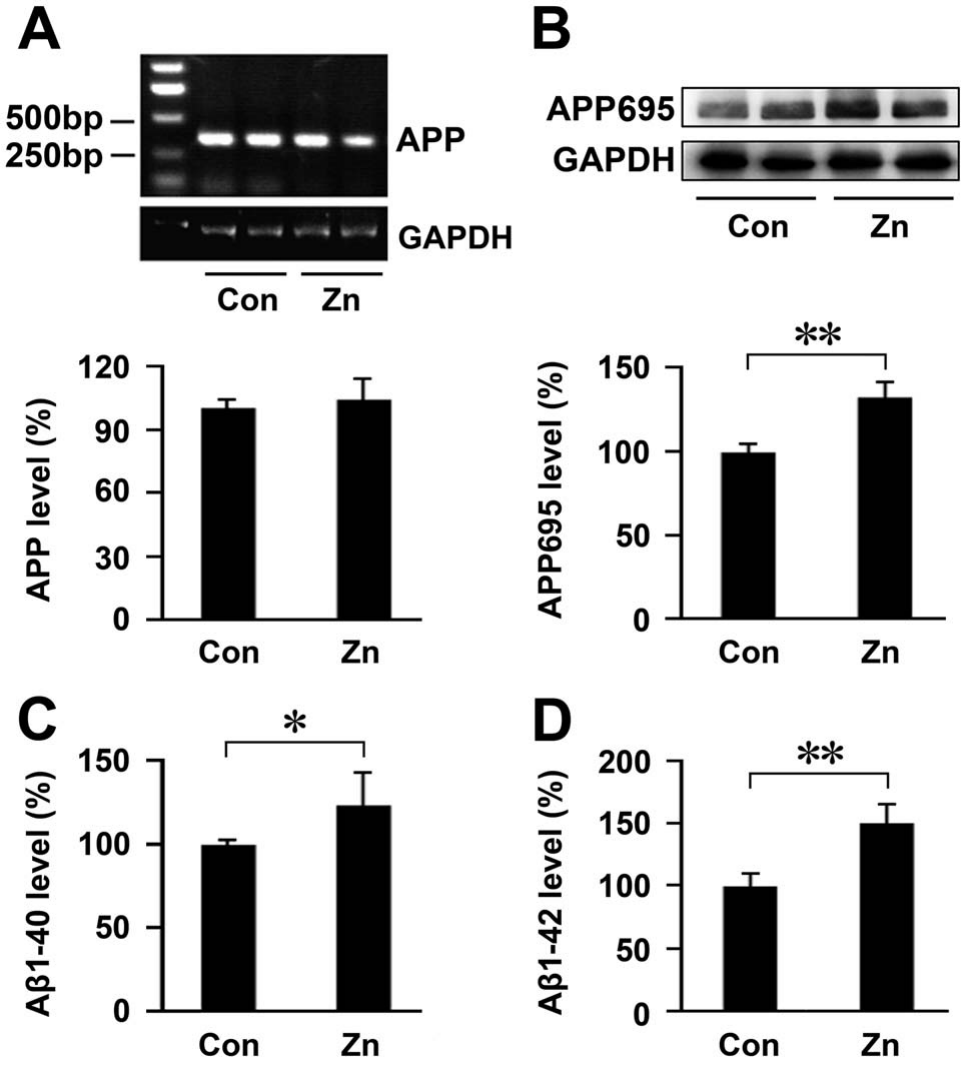
Expression level of APP and Aβ in APP/PS1 mice. (**A**) RT-PCR showed that no difference in APP mRNA levels between groups was detected in the transgenic mouse brain. GAPDH was used as an internal control. (**B**) Western blot analysis showed that high zinc treatment significantly increased the level of APP695protein. GAPDH was used as an internal control. (**C**, **D**) ELISA assay showed that the levels of Aβ1-40 and Aβ1-42 were significantly increased in Zn-treated mice compared with controls. * *p*<0.05, ** *p*<0.01 versus control group (Student's *t* test).

To further test whether a high dose of dietary zinc affects APP expression, the levels of APP mRNA and APP695 protein were measured by RT-PCR and Western blotting, respectively. As can be seen in Figure 5A, the expression levels of APP mRNA were not significantly changed between groups after examining brain samples of APP/PS1 mice treated with zinc or fed a normal diet. Western blot analysis revealed that zinc treatment significantly increased the level of APP695 protein by 132.79±16.82%, compared with controls fed a normal diet (Figure 5B).

To determine whether a high zinc intake had altered brain Aβ levels in APP/PS1 mice, a Sandwich ELISA for the detection of Aβ was employed (Figure 5C, D). Statistical analysis showed that a high zinc diet significantly increased the level of Aβ1-40 (*p*<0.05) and Aβ1-42 (*p*<0.01) in the brain, compared with controls. The levels of Aβ1-40 and Aβ1-42 were increased by 122.19±20.60% (Figure 5C) and by 148.96±15.67% (Figure 5D) in the brain homogenates of APP/PS1 mice with a high level of zinc in their drinking water, compared with controls fed a normal diet.

### High level of zinc in drinking water accelerates APP processing in the APP/PS1 mouse brain

**Figure 6:**
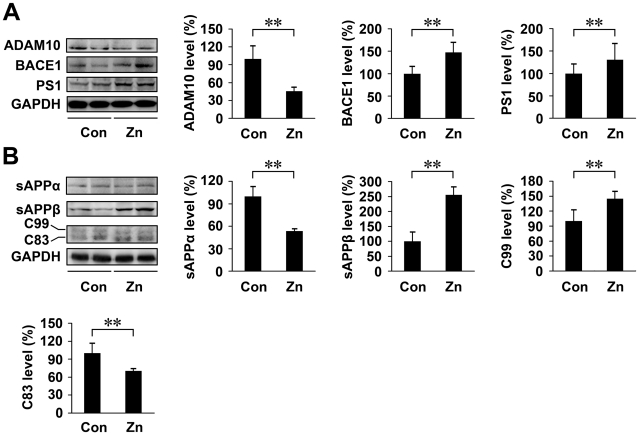
Expression level of APP cleavage enzymes and products in APP/PS1 mice. (**A**) The expression levels of ADAM10, BACE1 and PS1 in transgenic mouse brain were determined by Western blot analyses. GAPDH was used as an internal control. The level of ADAM10 was markedly reduced, whereas the level of BACE1 was significantly increased in Zn-treated mice, compared with controls. (**B**) Zinc treatment significantly reduced the level of sAPPα and C83, and increased the level of sAPPβ and C99 compared with the control group. ** *p*<0.01 versus control group (Student's *t* test).

To further examine whether a high intake of dietary zinc altered APP processing, the relative key enzymes (including ADAM10, BACE1 and PS1) and cleavage fragments of APP (including sAPPα, sAPPβ, C83 and C99) in the brain samples of APP/PS1 mice were subjected to Western blot analyses. As shown in Figure 6A, zinc treatment significantly decreased the expression level of ADAM10 by 45.80±7.01% compared with the control group (*p*<0.01; Figure 6A). In contrast, the level of BACE1 was significantly increased in zinc-treated mouse brain by 147.49±21.91% (*p*<0.01; Figure 6A). Furthermore, the level of PS1 in zinc-treated mouse brain was significantly increased by 130.44±36.80%, compared with the control group (*p*<0.01; Figure 6A).

We then examined the levels of α-secretase-generated sAPPα/C83 and β-secretase-generated sAPPβ/C99 fragments in the transgenic mouse brain. Zinc treatment significantly reduced the level of sAPPα by 53.55±3.32% (*p*<0.01; Figure 6B), and increased the level of sAPPβ by 255.62±27.24%, compared with the control group (*p*<0.01; Figure 6B). The level of C83 fragments was reduced by 70.48±4.27% and the level of C99 was increased by 144.65±15.79% in brain of zinc-treated mice relative to the controls (*p*<0.01; Figure 6B).

Taken together, these results indicate that the α-secretase cleavage activity is markedly reduced while β- and γ-secretase are increased in the brain of zinc-treated transgenic mice.

### High zinc exposure enhances amyloidosis in APPsw transfected cells

**Figure 7:**
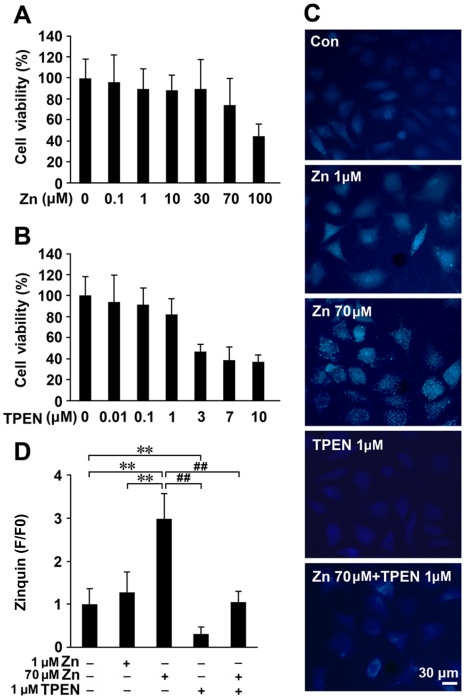
Cell viability and zinc accumulation in APPsw cells treated with zinc and TPEN. (**A**, **B**) MTT analyses were performed on the SHSY-5Y cells stably transfected with human APPsw, to select appropriate concentrations of zinc and TPEN for the *in vitro* studies. The cells were treated with indicated concentrations of zinc and TPEN for 8 h. Based on the cell viability, we chose a concentration of 1 µM ZnSO4 as “low zinc” and 70 µM as “high zinc” treatment, and 1 µM TPEN for zinc chelation treatment, respectively. (**C, D**) Zinquin fluorescence staining showing that zinc treatments enhanced the Zn-fluorescence accumulation, while TPEN reduced the density of fluorescence in APPsw cells. Fluorescence values were obtained during the period of basal conditions and the status at the end of each indicated administration. The y-axis data describe F/F0 fluorescence values. ** *p*<0.01 versus control group; ## *p*<0.01 versus 70 µM Zn treatment group (one-way ANOVA *Post hoc* Fisher's PLSD).

To further verify that high zinc exposure might be involved in APP processing and Aβ secretion, a human neuroblastoma SHSY-5Y cell line stably transfected with APPsw was used as an *in vitro* model [28], [29]. A zinc concentration of 1 µM (low zinc) and 70 µM (high zinc), and a concentration of TPEN (zinc chelator) of 1 µM were selected based on the evaluation of cell viability by MTT assay (Figure 7A, B). The zinc-specific fluorescent probe, Zinquin, was used to examine the levels of zinc in cells after zinc or chelator treatments. The results showed that Zinquin fluorescence was distributed in a punctate pattern in APPsw cells (Figure 7C). Zinc treatments increased the Zinquin fluorescence, while TPEN reduced it (Figure 7C). The fluorescence density assays showed that 70 µM zinc treatment significantly enhanced the Zinquin fluorescence by 300.31±56.19% (*p*<0.01; Figure 7D), whereas TPEN treatment reduced the fluorescence density by 30.94±14.65% compared with the control cultures (*p*<0.01; Figure 7D).

**Figure 8:**
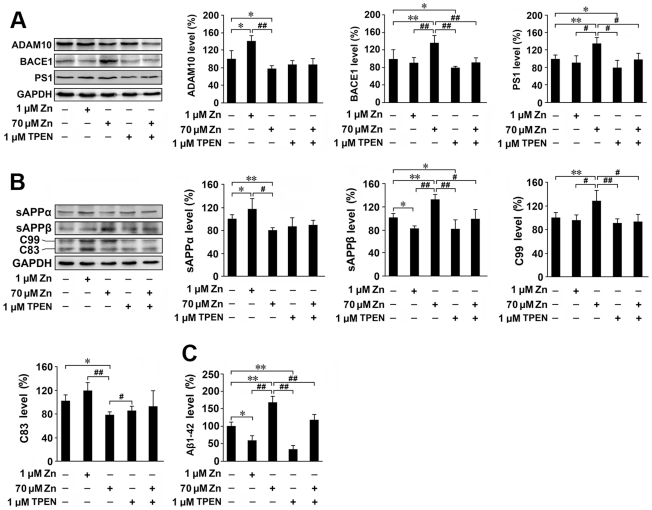
Expression level of APP cleavage enzymes and products in APPsw cells. SHSY-5Y cells stably overexpressing APPsw were exposed to 1 µM zinc, 70 µM zinc, 1 µM TPEN, and 70 µM zinc plus 1 µM TPEN, respectively, for 8 h. (**A**) Western blot was performed to determine the expression levels of APP cleavage enzymes, including ADAM10, BACE1 and PS1. GAPDH was used as an internal control. Low (1 µM) and high zinc (70 µM) treatment showed different effects on the expression of ADAM10. ADAM10 was markedly increased after low zinc treatment, but significantly reduced after high zinc treatment. There were no significant changes in ADMA10 levels in the TPEN or Zn + TPEN group, compared with controls. High zinc (70 µM) treatment significantly increased the levels of BACE1 as well as PS1. TPEN treatment reduced the BACE1 and PS1 levels. In Zn + TPEN group, the levels of BACE1 and PS1 were significantly reduced compared with the high zinc (70 µM) treatment group. (**B**) The expression levels of APP cleavage products, including sAPPα, sAPPβ, C83 and C99, were determined by Western blot analysis. GAPDH was used as an internal control. Low zinc (1 µM) exposure significantly enhanced the expression level of sAPPα, however, high zinc (70 µM) treatment markedly reduced the sAPPα expression level. There were no significant changes in sAPPα levels in the TPEN or Zn + TPEN treatment group compared with controls. The expression level of sAPPβ was significantly decreased after low zinc (1 µM) treatment, but was significantly increased after high zinc (70 µM) treatment. (**C**) ELISA results showed the Aβ1-42 level in the medium of APPsw cells following the indicated treatments. High zinc (70 µM) treatment significantly increased the levels of Aβ1-42, whereas low zinc (1 µM) and TPEN treatment reduced the levels of Aβ1-42. * *p*<0.05, ** *p*<0.01 versus control group; *# p*<0.05, ## *p*<0.01 versus 70 µM Zn treatment group (one-way ANOVA *Post hoc* Fisher's PLSD).

Consistent with our *in vivo* data from APP/PS1 transgenic mice, high zinc (70 µM) exposure significantly increased the APP cleavage enzyme levels of BACE1 by 135.50±17.05% and PS1 by 132.79±13.11% (*p*<0.01), and reduced the levels of ADAM 10 by 80.11±5.04% (*p*<0.05), respectively, in APPsw cells (Figure 8A). Subsequently, the levels of β-secretase-generated fragments sAPPβ and C99 were markedly increased by 131.46±8.57% and 126.95±22.46% (*p*<0.01), while the levels of α-secretase-generated sAPPα and C83 were decreased by 79.89±4.63% (*p*<0.01) and 76.94±5.61% (*p*<0.05), respectively, following 70 µM zinc treatment (Figure 8B). Also, chelation of zinc with 1 µM TPEN reversed the changes in the expression levels of the cleavage enzymes and fragments of APP (Figure 8B). Furthermore, ELISA detection showed that the levels of secreted Aβ1-42 in culture medium were increased by 166.27±23.04% in the 70 µM zinc treatment group (*p*<0.01; Figure 8C), and reduced by 37.18±10.36% with 1 µM TPEN (*p*<0.01; Figure 8C), compared with the control group. These data clearly indicated that high zinc exposure enhanced the amyloidogenic APP cleavage pathway and Aβ generation in APPsw overexpressing cells.

In contrast to high zinc exposure, low zinc (1 µM) treatment guided APP processing to the non-amyloidogenic pathway in our APPsw overexpressing cells. Following 1 µM zinc treatment, the levels of AMAM10, sAPPα and C83 were increased by 142.21±12.04% (*p*<0.05; Figure 8A), 116.71±19.07% (*p*<0.05; Figure 8B) and 117.81±12.47% (*p*<0.05; Figure 8B), whereas the levels of BACE1, sAPPβ and C99 were reduced by 89.61±13.34% (*p*<0.05; Figure 8A), 81.03±4.80% (*p*<0.05; Figure 8B) and 94.06±9.89% (*p*<0.05; Figure 8B), respectively, compared with the controls. The level of secreted Aβ1-42 in culture medium was decreased by 64.43±11.77%, following 1 µM zinc treatment (*p*<0.05; Figure 8C). These data are consistent with previous reports showing that exposure to low concentrations of zinc (≤50 µM) significantly enhances the levels of secreted APP and results in reduced release of Aβ in a medium of cultured CHO-K1 cells [30], [31].

## Discussion

Both APP and its proteolytic byproduct Aβ, which play central roles in senile plaque formation in the pathogenesis of AD, are zinc-containing metalloproteins that contain zinc-binding domains [32]. Therefore, it is rational to speculate that zinc overload may be involved in APP expression, Aβ generation and aggregation. In the present study, involving treatment with a high level of zinc in the drinking water of APP/PS1 mice, we found that mice fed a high zinc diet exhibited spatial learning impairments as shown by Morris water maze tests. Apart for body weight loss, fur color changes (data not shown), raised serum and brain zinc levels, and a high zinc content in the drinking water resulted in no other overt signs of toxicity such as general behavioral and neurological changes during the entire observation period which our model mice were given a high zinc diet. This is in agreement with previous reports showing that there was no serious toxicity in C57BL/6 mice after chronic zinc treatment at the same dose [33], [34]. Thus, we further evaluated the effects of a chronic high dietary zinc intake on accumulation of Aβ deposits, as well as APP expression and cleavage in the APP/PS1 transgenic mouse brain.

We and others have reported that zinc is highly concentrated in amyloid plaques in human postmortem brain samples [5], [6], [7], [8], [10], [18], [35] and in AD transgenic mouse brains [9], [36]. Here, we found that a high intake of dietary zinc resulted in an increase in zinc-containing plaques in APP/PS1 transgenic mice. Coincident with the AMG results, Aβ immunohistochemical analyses demonstrated that there was an increased Aβ burden in transgenic mice fed a high zinc diet. Since the small peptide Aβ possesses selective high- and low-affinity zinc binding sites [32], [37], and zinc at a concentration of 300 nM can rapidly destabilize Aβ and result in fibril formation [11], [37], it is likely that an overload of brain zinc increases Aβ binding and, hence, enhances Aβ aggregation and plaque formation in the brain after chronic administration of a high zinc diet.

Zinc is toxic and, besides its physiological roles, it is involved in neuronal and glial death through activation of multiple intracellular pathways leading to necrotic, apoptotic and autophagic neuronal death [38], [39], [40], [41]. The elevated level of zinc in the AD brain is caused, at least partly, by the abnormal distribution and expression of zinc-regulating proteins such as ZnTs and DMT1 [18], [19], [21]. At an early stage of AD, the elevated brain zinc results in the formation of zinc-Aβ complex, which is of some benefit in protecting against zinc toxicity [42], [43], [44]. On the other hand, recent studies have shown that soluble Aβ is a major factor in neuronal and synaptic pathology, since it is more toxic than insoluble Aβ [45], [46], [47]. It is likely that the initial zinc-Aβ complex and subsequent Aβ aggregation inhibits Aβ mediated neurotoxicity. However, it is worth noting that the initial zinc-Aβ complex may serve as a seed for the process of Aβ aggregation and plaque formation in the brain [5], [11], [48], [49]. Although it is still debatable whether Aβ aggregation mediated by interaction with zinc plays a role in reducing the toxicity of soluble Aβ or whether the zinc-containing plaques themselves are toxic to neuronal cells [44], [50], [51], [52], the interaction between Aβ and zinc seems to be a critical factor for activating AD pathological processes. Nevertheless, our present data suggest that a high zinc intake leads to more zinc-Aβ complex formation, accelerates Aβ deposition and enhances the amyloid burden. Further studies are needed to elucidate the paradoxical role of zinc in plaque pathology [31].

APP protein contains a novel zinc binding motif which is located between the cysteine-rich and negatively charged ectodomains [32]. Besides its structural role, zinc may be involved in the function and metabolism of APP protein, and produce an even greater deposition of Aβ. However, apart for several *in vitro* studies that tested the effects of zinc on APP processing [30], [53], there are no detailed reports whether zinc binding to APP alters APP processing and Aβ production in AD transgenic animal models. In the present study, we found that a high intake of dietary zinc significantly increases the expression levels of APP protein in APP/PS1 transgenic mouse brain. We also found that high-dose zinc treatment results in reduced expression levels of ADAM10, but enhances the levels of BACE1 and PS1, resulting in increased secretion of sAPPβ over sAPPα in the transgenic mouse brain. Further, consistent with our *in vivo* data, high zinc (70 µM) exposure suppresses α-secretase cleavage, but enhances β- and γ-secretase cleavage of APP and Aβ generation in APPsw overexpressing cells. Thus, our *in vivo* and *in vitro* studies clearly show that high-dose zinc treatment enhances the amyloidogenic APP cleavage pathway and Aβ secretion. Interestingly, a recent study involving APP/PS1 mice fed a zinc-deficient diet has shown that such a diet increases the plaque volume but does not alter the total plaque number in the brain [27]. Chronic high zinc- or copper-treated mice overexpress APP-C100, which contains Aβ but not the N-terminal zinc and copper binding domain of APP, resulting in reduced soluble Aβ levels but with no changes in the total Aβ levels in the brain [54]. It has also been reported that exposure to copper and, presumably, a mixture of other metals in drinking water results in enhanced Aβ deposition in the brains of rabbits fed a high cholesterol diet [55]. Alain Boom and colleagues showed that 100 µM zinc induced the appearance known to be associated with increased tau phosphorylation, suggesting that zinc plays a considerable role in the development of tau pathology associated to Alzheimer's disease [56]. On the other hand, some reports have shown the protective effects of low micromolar concentrations of zinc against Aβ cytotoxicity [43], [44], [57]. So far, the role of zinc in AD remains debatable. Both high and low zinc could play a harmful role. Whether or not to supply zinc and what the suitable dose range should be are topics worthy of future research on AD. Taking these findings together with the present evidence that high-dose zinc treatment leads to enhanced amyloidogenic APP cleavage and Aβ aggregation in the APP/PS1 mouse brain and APPsw overexpressing cells, it can be concluded that disturbed metal homeostasis is involved in multiple steps of APP processing and Aβ deposition by a series of complicated mechanisms.

In summary, the present study provides evidence that chronic exposure to high zinc levels in drinking water leads to an increase in APP expression, amyloidogenic APP cleavage and Aβ deposition in the APP/PS1 transgenic mouse brain. The present data, together with previous reports, suggest that excess zinc exposure could be a risk factor for AD pathological processes, and corrections of metal abnormalities in the brain are beneficial strategies for AD prevention and therapy.

## Materials and Methods

### Ethics statement

The experimental procedures were carried out in accordance with the regulations of the animal protection laws of China and approved by the animal ethics committee of China Medical University (JYT-20060948). All efforts were made to minimize animal suffering and the number of animals used.

### Animals and Treatments

Male APP/PS1 double transgenic mice (B6C3-Tg (APPswe, PSEN1dE9) 85Dbo/J mice) were used in the present study (breeding pairs were obtained from the Jackson Laboratory, West Grove, PA). They were kept in cages in a controlled environment (22–25°C, 50% humidity). Mice at the age of 3 months were randomly assigned to one of two groups (n = 12 in each group). (1) Control group: mice were given a standard diet (30 ppm zinc) and deionized water *ad libitum*. (2) Zinc group: mice were given a standard diet and deionized water containing ZnSO_4_ (20 mg/ml). The doses of zinc chosen for this study were based on previous reports showing that it produced no serious toxicity in C57BL/6 mice after chronic treatment [33]. The potential toxicity of high-dose oral zinc on transgenic mice was evaluated by monitoring their general aspect and body weight. As reported previously [33], [34], apart for a coat color change from black to bright brown on the back, and a decline in body weight starting from the 14th week after zinc treatment, no other overt signs of toxicity, such as general behavioral and neurological changes, were observed in mice on a high zinc diet during the entire observational period. At the age of 9 months, blood samples were drawn from the heart just prior to decapitation, and the levels of zinc in serum and brain were analyzed using a polarized Zeeman atomic absorption spectrophotometer (Hitachi 180-80, Japan).

### Morris Water Maze

Morris water maze tests were carried out as previously described with few modifications [58], using a circular tank, equipped with a digital pick-up camera to monitor the animal behavior and a computer program for data analysis (ZH0065, Zhenghua Bio-equipments, China). Briefly, one week before reaching the age of 9 months, transgenic mice were trained for 2 days to remember the visible platform, which is placed in the center of the northwest quadrant in the tank with opaque water. From the 3rd to 7th day, the platform was placed just below the water surface (hidden platform) for the place navigation test, and each mouse was subjected to 3 trials per day with an inter-trial interval of 1 min. For each trial, the latency to escape to the hidden platform and the path length were recorded. At the 8th day, the platform was removed from the tank for the probe trial. The number of times the animal crossed the center of the northwest quadrant at an interval of 1 min was recorded. Finally, data for the escape latency, the path length and the number of passing times between groups were analyzed statistically.

### Tissue Preparation

The day after the Morris water maze tests, mice were anaesthetized with sodium pentobarbital (50 mg/kg, i.p.) and sacrificed by decapitation. The brains were removed immediately and split sagittally into halves. The left hemisphere was kept at −80°C for Western blotting, RT-PCR or ELISA analyses. The right hemisphere was further cut into two slabs. One was immersed in 3% glutaraldehyde for zinc autometallographic analysis while the other was placed in 4% paraformaldehyde for immunohistochemical staining.

### Autometallography and Stereological Assessment

AMG was performed to analyze the distribution of zinc in the transgenic mouse brain according to our previous reports [20], [59]. In brief, brain slices (2 mm) were cut with a vibratome and immersed in a mixture of 3% glutaraldehyde and 0.1% sodium sulfide at 4°C for 2 days. The slices were placed in 30% sucrose, frozen with liquid nitrogen and 30-µm cryostat sections were prepared. Brain sections containing typical hippocampal structures from 2 groups were selected, placed in the same jar, and incubated in AMG developer at 26°C for 60 min. The AMG development was stopped by rinsing with 5% sodium thiosulfate for 10 min. After several washes with distilled water, the sections were dehydrated, covered with neutral balsam and examined with a light microscope equipped with a digital color camera. The sodium diethyldithiocarbamate trihydrate (DEDTC, Merck, 6689) control procedures were performed to ensure the specificity of the zinc ion staining [59].

The stereological assessment of zinc load in the AMG-stained brain sections was performed as reported previously [18], and this was carried out in matched brain areas from different groups. Briefly, a set of every 6th systematically sampled 30-µm-thick AMG-stained sections of the transgenic mouse brain (yielding typically 5 sections/mouse) was selected. The total number of zinc-positive plaques was counted using the optical fractionator technique, while the area of zinc-positive plaques was analyzed in the same sections using the area fraction technique. The data were analyzed with Image-Pro Plus 6.0 software (Media Cybernetics, USA).

### Immunohistochemistry and Aβ Load Measurements

Routine free floating ABC procedures were applied. Cryostat sections (30 µm) were treated with 3% hydrogen peroxide (H_2_O_2_) in PB for 10 min to reduce endogenous peroxidase activity. After rinsing, the sections were treated with 5% bovine serum albumin and 3% goat serum in TBS for 1 h. They were then incubated with mouse anti-Aβ (1∶500, Sigma, A5213) overnight at 4°C. After rinsing in TBS, the sections were incubated with 1∶200 diluted biotinylated goat anti-mouse IgG for 1 h at room temperature (RT). The sections were then rinsed and treated with an ABC kit for 1 h at RT. The sections were rinsed in TBS and incubated with 0.025% 3, 3-diaminobenzidine (DAB) plus 0.0033% H_2_O_2_ in TBS for 10 min. After rinsing, the sections were dehydrated, covered with neutral balsam, and examined with a light microscope. Control sections were incubated with normal serum instead of Aβ antibody followed by all subsequent incubations as described above. The stereological assessment of Aβ load in the Aβ-immunostained brain sections was performed according to the above described procedure.

### Cell Culture and Drug Treatment

Human neuroblastoma SHSY-5Y cells stably transfected with human APPsw [28], [29] were grown in DMEM supplemented with 10% heat-inactivated fetal calf serum, 100 U/ml penicillin, 100 µg/ml streptomycin and 200 µg/ml G418 at 37°C in humidified 5% CO_2_ air. Cells were incubated in serum-free medium for 2 h when they reached nearly 70% confluence. Then, cells were incubated with ZnSO_4_ (1 µM, 70 µM), TPEN (1 µM) or ZnSO_4_ (70 µM) plus TPEN (1 µM) for 8 h. The concentrations of zinc and TPEN were selected based on routine MTT assay. To analyze the zinc entry into the cells, the staining with a zinc-specific fluorescent dye, Zinquin, was carried out by incubating the cells with 0.24 µM Zinquin ethyl ester (Alexis, USA) for 30 min, and then examining them under a fluorescent microscope equipped with an image analysis system.

### Western Blotting

The preparation of lysates from the transgenic mouse brain samples and culture cells, and the Western blots were performed as described previously [19], [21]. Briefly, samples were homogenized in an ice-cold lysis buffer. Each homogenate was centrifuged at 12,000 rpm for 30 min at 4°C, the supernatant was collected and the total protein levels were measured using a BCA protein assay kit. Proteins (50 µg) were separated on 8–15% SDS polyacrylamide gels according to the molecular weight of the detection proteins, and transferred onto PVDF membranes using an electron transfer device (45 V, overnight at 4°C). The membranes were blocked with 5% non-fat milk in TBS containing 0.1% Tween-20 for 1 h and then incubated with a primary antibody for 2 h at room temperature. The primary antibodies used were: rabbit anti-ADAM10 (1∶1000, Millipore, AB19026), rabbit anti-APP695 (1∶4000, Chemicon, AB5352), rabbit anti-BACE1 (1∶1000, Sigma, B0681); goat-anti-PS1 (1∶1000, Millipore, MAB1563); mouse anti-sAPPα (1∶500, IBM, 2B3, JP11088); mouse anti-sAPPβ (1∶500, IBM, 6A1, JP10321); rabbit anti-APP-CTFs (1∶4000, Sigma, A8717), and mouse anti-GAPDH (1∶10000, KC-5G5, Kang Chen, 0811). Bound secondary antibodies were visualized using an enhanced chemiluminescence kit (Pierce, CA). Blots were repeated at least three times for every set of conditions. The band intensities were quantified using Image-pro Plus 6.0 analysis software.

### RT-PCR

Total RNA was isolated using Trizol reagent (Invitrogen) after homogenizing the brain tissue samples. The isolated RNA was examined by UV-spectroscopy at 260 nm. Total RNA (2 µg) of each sample was first transcribed to cDNA using a Reverse Transcription System kit (Promega, Madison, WI, USA). PCR amplification was performed with reagents from Promega. The cDNA solution was amplified with primers based on the human APP sequences. The primer sequences were: APP: 5′-GACTGACCACTCGACCAGGTTCTG-3′ (upstream), 5′-CTTGAAGTTGGATTCTCATACCG-3′ (downstream); GAPDH: 5′-ACGGATTTGGTCGTATTGGG-3′ (upstream), 5′-CGCTCCTGGAAGATGGTGAT-3′ (downstream). Amplification was performed as follows: APP: 35 cycles of 95°C for 30 s, 62°C for 30 s and 72°C for 30 s; GAPDH: 30 cycles of 95°C for 45 s, 58°C for 45 s, and 72°C for 60 s. The PCR products were normalized in relation to standards of GAPDH mRNA. The results were determined and quantified with ChemDoc XRS Quantity One software.

### ELISA

Aβ levels were determined using ELISA assay as described previously [21]. In brief, samples were placed in a 1∶8 dilution of 5 M guanidine HCl/50 mM Tis HCl and thoroughly ground. The homogenate was diluted with dilution buffer containing an inhibitor protease complex and centrifuge at 12,000 rpm for 30 min at 4°C. The samples were then loaded on to 96-well plates and the level of Aβ was determined using ELISA kits for Aβ1-40 (Kuregen, KU0821E-10) and Aβ1-42 (Invitrogen, KHB 3441), according to the manufacturers' protocols. The absorbance was recorded at 450 nm using a 96-well plate reader.

### Statistical Analysis

Data are expressed as means ± SEM. For water maze analysis of latencies and path length, repeated measures analysis of variance (ANOVA) were performed, and differences among means were evaluated with multivariable ANOVA. Other comparisons were analyzed by Student's *t* test, and one-way ANOVA *Post hoc* Fisher's PLSD for cultured cells. All data were analyzed using SPSS 13.0 software, and statistical significance was assumed if *p*<0.05.
